# Caries of Superior Maxillare and Death

**Published:** 1846-12

**Authors:** 


### Collectanea.

*Caries of Superior Maxillare and Death.*—CASE 1.—William S ——, aged fifty, was admitted under Dr. Taylor, physician to University Hospital, April 28th, 1846, of short stature, and moderately developed frame; complexion dark, and until lately, healthy and florid; temperament sanguine. He is a gas-fitter, for forty years continuously in employ; of late well-fed and clothed, but much exposed to wet and cold; irregular in his habits at times. Before the present illness, robust, having had no illness since a slight attack of rheumatism twenty-two years ago.

About nine weeks since, without any previous indisposition or assignable cause, his mouth became sore, and his face swollen, especially over the right superior maxilla; the swelling has since continued, though now it is less in degree. Three weeks ago matter began to flow from his nostrils; none of it passed into his mouth. He left his work partially five weeks ago, but not entirely, until the last fortnight. He has only kept his bed during the last three days.

He is now emaciated, with much prostration; complexion pale, very sallow and muddy; countenance very anxious; expression and general aspect so much altered, that he is irrecognisable by his master; lips pale and dry; sordes on the teeth; breath fœtid, apparently so from the affection of the jaw; no appetite; bowels open daily. The left leg, from the ankle to the knee, is considerably swollen, pits on pressure, and there is a little redness of the skin at the back; pain in the leg, increased on pressure, the induration greatest along the outer side; no hardness in the course of the vessels; no swelling of the right leg, or induration, swelling, or tenderness of the left thigh; superficial veins of the legs, especially of the left leg, enlarged. Sounds and impulse of the heart healthy; pulse 104, very small and weak. Pain in the right side of the chest, not felt until he was carried up into the ward; breath short for the last fortnight; little, if any cough; respiration chiefly costal; position of lying frequently altered; difficulty of articulation; some tumor of tongue and limbs. On percussion in front, the infra-axillary region sounds duller on right side than on left; dullness over the region of the liver extending upwards to the fifth rib, and a very little below the ribs; the right side under the clavicle duller than the left. Respiration pretty distinct under the right clavicle; lower down, very feeble; about the mamma, a little bronchial, though still feeble; low down, on the right lateral surface, a friction-sound. Behind, on percussion, the right side is generally duller than the left, especially in the middle third, where there is some scattered, large, deep-seated crepitation; in the upper part of the right side, and generally on the left side, the respiration is more healthy. Ordered beef-tea, milk, and four ounces of wine in twenty-four hours.

April 29th.—Pulse 100, very small; pain in the right side. Behind, over the lower third of the right side, is a modification of friction-sound resembling that produced by rubbing a wetted finger on glass; in the middle third, respiration bronchial, with some large crepitation, which again is not so well heard in the upper third; on the left side, respiration tolerably natural. On percussion, the right side dull generally. No delirium during the night; more pain now on the right side; urine clear, scanty, specific gravity 1016, slightly acid, contains albumen, which, when coagulated, occupies one-sixth of its bulk. To have every four hours, (conditionally,) potassio-tartrate of antimony, a quarter of a grain; peppermint water, one ounce. 12 P. M.—Pulse scarcely perceptible. Omit the medicine.

30th.—Left leg less swollen; pulse 96, small and weak; skin moist, tongue dry. There is a blush of redness and swelling about the nose; breath very fœtid. On the right side of the chest some sonorous rhonchus, and (it may be) some modified friction-sound; other physical signs much as yesterday. To have eight ounces of port wine in twenty-four hours.

May 1st.—He was delirious last night. The redness, &c. of the nose remains; left leg still swollen; pulse 120, weak, but not weaker than yesterday; abdomen rather large and tympanitic. In the chest, in front, the right side generally duller on percussion than the left; respiration somewhat puerile under the clavicle; in the lower half of the right side very feeble, somewhat bronchial. Behind, in under third of right side, bronchial, with large crepitation, and some friction-sound. In the left lung, much muco-crepitant rhonchus; tongue very dry and brownish. Wine as before, and increased to twelve ounces. Ten P. M.—The wine increased to sixteen ounces in the twenty-four hours. An enema was returned without any fœces. There is a large hæmorrhoidal tumor outside of the anus which did not exist two days ago; aspect more prostrated; breathing more laborious; mouth open; skin perspiring; pulse 120, soft and weak, but not more so than yesterday; tongue dry, with a black fur; sordes on teeth; urine passed in the bed; breath less fœtid. There are a few spots like the rose spots of fever on the chest. Wine ordered freely; quantity not limited. He gradually sank, and died at half past four in the afternoon.

*Post-mortem examination forty-six hours after death; fine weather.*—There is still some swelling of the left leg, the cuticle raised by effusion; moderate emaciation; green lines in the course of the superficial veins. About two drachms of serum in each lateral ventricle of brain; cerebral substance soft. The right pleura contained above fifteen ounces of turbid serum; old adhesions opposite third, fourth, and fifth ribs, on the lateral surface near the nipple; other old adhesions near the spine; pleura costalis more vascular than usual; a little pus, on pleura pulmonalis, and a little very soft lymph on the lower and middle lobes of lungs, which weighed twenty-four ounces and a half, and contained much less air than usual. Neither upper or lower lobe is very brittle, both are fleshy, the lower very dark; mucous membrane of bronchi not remarkably red. In the left pleura, five ounces of fluid, deeply tinged with blood. The left lung, weighing twenty-nine and a half ounces, was larger than the right, and contained more air; the upper lobe is considerably puckered, and has in it several distinct portions of putty-like matter; the lower lobe contained bloody serum, very little frothy; it is brittle, and has an odor of putrefaction; mucous membrane redder than in right lung, uniformly so, and apparently from imbibition. The pericardium contained nothing to be noticed except a patch over the right ventricle. Heart exceedingly soft and flabby, uncontracted, in other respects healthy; blood in right ventricle nearly all fluid, with some small air-bubbles; weight, nine and a half ounces; lining membrane of heart and of aorta deeply colored by imbibition. Mucous membrane of stomach softened at the cul de sac, softened and mamelonated near the pylorus. At the lower part of the ileum, one of Peyer's patches rather distinct; large intestines much distended with gas; intestines and mesenteric glands otherwise healthy. Spleen about six inches in length; its consistence for the most part not greater than that of cream; weight, seven and a half ounces. Liver full-sized; above of a deep blue; on a section for the most part pale and very soft; the blood in it contains some air. The gall-bladder contains very thin yellow bile, and a number of calculi which sink in water and are very brittle. The right kidney is much enlarged, weighing seven ounces: much of its outer surface distinctly red, in red punctiform spots; substance flabby and very soft; the redness greater in the cortical than the tubular portion. The left kidney is smaller, but presents the same characters in a less degree. The urinary bladder a little distended. The veins of the left leg and the left knee-joint did not contain pus. Extensive caries of the superior maxillary bone.

*Summary of remarks by Dr. Taylor.*—On admission, his countenance was expressive of suffering and anxiety rather than of pain, and the general symptoms at first suggested a fever of a typhoid type. The next thing which arrested attention was the state of the left leg; this evidently depended on some local cause, which might have been phlebitis, but of this there was not distinct evidence. The dyspnœa which had existed for a fortnight, and the signs discovered on examining the chest, made it probable that there was a pneumonia, with some pleurisy, rather than a mere pleuritic effusion; and when it was found that the urine contained albumen, it was inferred that the patient labored under secondary diseases dependent upon Bright's disease, the question still remaining undecided as to the existence of phlebitis. However, it was obvious from the general symptoms, that the patient required support, and wine, beef-tea and milk, were given, at first cautiously and conditionally. After this, chiefly in deference to received opinion, tartar emetic was ordered, but only conditionally, and its effects being too depressing, it was discontinued in the evening. The rose spots during the last days again suggested that the case might be really typhus; but this idea was again set aside by the other symptoms; and, indeed, these spots are not absolutely pathogmonic of typhus, but occur in other diseases of low type. The signs of putrefaction at the inspection were disproportioned to the period after death at which it was made. No evidence of phlebitis being then found as is frequent in the veins and joints, we may ascribe the appearances in the left leg to some inflammation of an erysipelatous kind, itself dependent on a more general disease. The right pleura contained distinctly the products of inflammation, so that the effusion was not merely dropsical.

But all the signs were in accordance with what we find in pneumonia more frequently than in pleurisy, and even when you are most upon your guard, the diagnosis of effusion is not so simple as authors represent it to be. The kidneys increased in size, their texture softened, and reddened from evidently increased vascularity, I believed were acutely inflamed, and the microscope afterwards greatly confirmed this. During life this was only indicated by the albuminous urine, (and we had the character of Bright's disease in the urine without that disease being present;) there was no pain to indicate this inflammation, which thus agreed with the others. Most of the symptoms and the post mortem appearances belong to that class of general diseases which Rayer has shown to depend upon the absorption of a morbid poison; in which there exists a disposition to acute inflammations, with little or no pain, a typhoid condition, a tendency to early putrescency, &c. He has traced them as induced by gangrene, charbon malign, and purulent absorption, &c., and he designated the disease from the general affection of the kidney, nephritis from morbid poison. Considering this and the other inflammations secondary to the presence of the poison in the blood, the erysipelatous inflammation in the leg would result from the same cause; but we approach a more speculative region, when we come to consider what the morbid poison was in this case. There is not sufficient to indicate that it was that of ordinary typhus, or of phlebitis, and if not one of these, the only cause I can suggest, and the one to which I confess I lean, is the absorption of putrid matter from the diseased bone;—we know that such absorption of gangrenous matter is adequate to produce such effects. With this view of the pathology, what I said before of the treatment is fully corroborated, and not regarding the secondary inflammation as ordinary acute disease, experience has proved that our only resource as to general treatment is, the free administration of wine and other support.—*London Lancet.*

